# Optimizing the incubation conditions of third-stage larvae of the camel nasal bot *Cephalopina titillator* (Diptera: Oestridae) for harvesting adult flies

**DOI:** 10.14202/vetworld.2024.2322-2328

**Published:** 2024-10-17

**Authors:** Mohammad Nafi Solaiman Al-Sabi, Hams Almohammed, Fatema Alghatam, Ghadeer Alhafiz, Omar Al-Jabr, Ahmed M. A. Meligy

**Affiliations:** 1 Department of Basic Veterinary Medical Sciences, Faculty of Veterinary Medicine, Jordan University of Science and Technology, P. O. Box 3030, Irbid 22110, Jordan; 2 Department of Microbiology, College of Veterinary Medicine, King Faisal University, P.O. Box 400, Al-Ahsa 31982, Saudi Arabia; 3 Department of Clinical Sciences, Central Lab, College of Veterinary Medicine, King Faisal University, P.O. Box 400, Hofuf, Al-Ahsa 31982, Saudi Arabia; 4 Department of Physiology, Agricultural Research Center (ARC), Giza, Egypt

**Keywords:** camel nasal bot, *Cephalopina titillator*, eclosion success, *in vitro* incubation, life span, myiasis

## Abstract

**Background and Aim:**

Understanding the developmental conditions of *Cephalopina titillator* larvae and their effect on the success of pupation and adult emergence can help prevent and control this disease in camels. Incubating *C. titillator* larvae *in vitro* requires optimized conditions that have not been adequately reported in the literature. This study aimed to optimize conditions for harvesting adult flies from third-stage larvae (L3).

**Materials and Methods:**

L3 collected from naturally infested camels was washed in sterile saline, weighed, and placed in vials containing local sand. The vials were covered with gauze and incubated at 30°C–36°C with 60%–62% relative humidity in an environmental chamber.

**Results:**

A minimum critical weight of 754 mg per larva was found to be essential for the successful eclosion of the adults, regardless of the sex of the emerged flies. The pupariation period lasted 1–8 days (d) and took 5–13 days. Most incubated L3 formed puparia, but most failed to emerge as adults. The success rate of eclosion was 37.1%. The hatched adults survived for up to 18 days, and the females survived longer (12 d; 6–18) than the males (8.1 d; 3–16).

**Conclusions:**

The higher eclosion success tendency of certain sand types might be influenced by the sand’s physical and/or chemical characteristics. The current *in vitro* conditions resemble those during the hot seasons and are suitable for harvesting viable adults of *C. titillator* from L3.

## Introduction

The larvae of the Dipteran fly *Cephalopina titillator* inhabit the nasal cavities and nasopharyngeal regions of camels wherever they exist [1–3]. Gravid females of *C. titillator* hover in front of the nostrils of camels and deposit eggs in clusters [4] or larvae on the nasal openings [5]. The larvae of *C. titillator* undergo a prolonged period of growth inside the nasal cavity and nasopharynx of camels for up to 9–11 months. During this time, the larvae feed on the epithelial cells of the mucosa of the nasal cavity and nasopharynx and molt inside the host from the first-stage larva (L1) to the third-stage larva (L3), which may reach an average length of 2.5 cm. Several larvae can be found in an infested camel, leading to inflammation of the epithelial linings and serous discharges stained with blood [4–7]. Infested camels also suffer from distress, which affects their behavior and feed intake [4]. Such infestations can be treated using various medications, including herbs and oils [8]. The diagnosis of *C. titillator* is challenging in itself [9–11]; nonetheless, camel owners, due to economic and/or educational reasons, rarely implement these practices.

Efforts to control adult *C. titillator* flies on a national or wide scale are challenging and seldom, if any time, have been performed. Flies are characterized by having a short life span in nature, a maximum of 3 weeks, due to their rudimentary and non-functional mouthparts [4, 5]. Accordingly, to perpetuate the life cycle in nature by producing new larvae, females and males have limited time to locate each other and mate in nature after their emergence from the pupa. On the other hand, studying the biology and life habits of adult flies *in vitro* requires capturing a considerable number of these flies from nature, which might seem impossible due to their short life expanse [12, 13]. Alternatively, studying the biology of these flies can be achieved by incubating the larval stages *in vitro* to harvest the adults. This requires determining a favorable environment for larval growth to maximize the success rate of adult flies eclosion from the pupae, but unfortunately, the literature lacks detailed descriptions of controlled experiments that indicate favorable *in vitro* conditions for larval incubation. The described *in vitro* methods in the literature that are used for cultivating myiasis-producing flies are generally those dedicated to maintaining dipteran maggots of the Calliphoridae family, which usually inhabit their hosts for few days [14–17], not those of the Oestridae family, which inhabit the host for months.

Several studies have indicated the occurrence of *C. titillator* in camel year-round and larvae occasionally pupate on hot sand to complete their life cycle [18, 19]. Accordingly, it is expected that larvae of *C. titillator* will tolerate raised temperatures in the laboratory for pupation and adult development. However, information on the developmental conditions of *C. titillator* larvae and their effects on the success of pupation and adult emergence are lacking in the literature. Therefore, this study aimed to: (1) optimize the *in vitro* incubation conditions of the third-stage larvae of *C. titillator* (L3) using different substrates and ventilation directions on the incubation vials, (2) determine the critical initial weight of larvae necessary for successful eclosion, (3) measure eclosion success, determine the sex ratio of emerged flies, and determine the life span (longevity) of the flies in the laboratory, and (4) measure the reduction in weight of adult flies from emergence to death in the laboratory. The outcomes of this study should widen our knowledge of the biology of the obligate myiasis-producing oestrid.

## Materials and Methods

### Ethical approval

The maggots used in this study were collected from dead camels from a local abattoir; hence, no ethical approval was needed in this situation.

### Study period and location

This study was conducted from September 2020 to October 2021. The samples were collected from the local slaughterhouse in the Al-Omran district of Al-Ahsa City, Saudi Arabia.

### Collection and processing of L3

**Figure-1:**
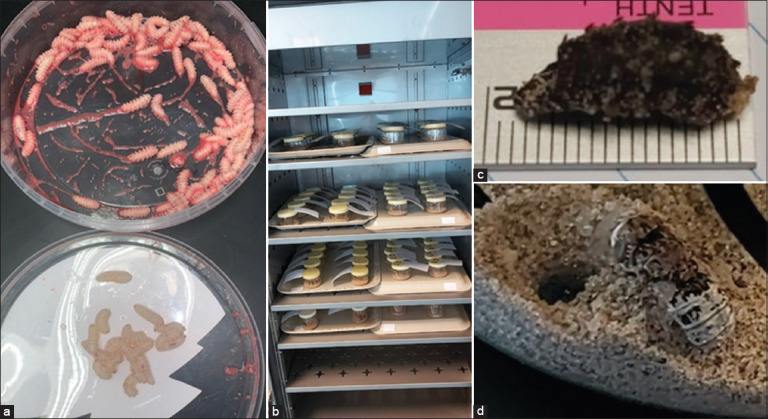
Methodology of incubating third-stage larvae of *Cephalopina titillator* (L3). (a) Freshly recovered L3 show blood staining before (top) and after washing (bottom). (b) Experimental vials incubated in the environmental chamber. (c) Measurement of larval dimensions. (d) Adult *C. titillator* emerging from the sand ground.

Third-stage larvae were manually collected from the nasopharynx of naturally infested adult camels of local breeds from the local slaughterhouse in the Al-Omran district of Al-Ahsa City, Saudi Arabia. The collected larvae were placed in an empty-dry polystyrene transport box or plastic containers (Figure-1a, top) and transported within 1-h post-collection to the Entomology Lab of the College of Veterinary Medicine, King Faisal University (Vet-KFU). In the laboratory, L3 was washed with 50ml of sterile saline (0.9% w/v Sodium Chloride, Intravenous Infusion BP, Sterile Non-pyrogenic, B. No.: 136857, Pharmaceutical Solutions Industry, Jeddah, Saudi Arabia) and then poured into a vial containing larvae. The vial was then immediately shaken for 2 s, and saline was poured off into a plastic container with holes in its bottom to retrieve the washed L3. Once all the water had dripped out, the larvae were placed on clean and dry tissue for a few seconds. Only larvae that showed active motility for at least 2 min were included in the study (Figure-1a, bottom).

### Effect of substrate type and ventilation direction on vial eclosion success

Choosing sand as a substrate for incubating L3 was based on the results of a previous study by Bekele [20], who successfully reared adult L3 of *C. titillator* in containers with local sand as a substrate. Here, the sand substrates were collected from two nearby desert locations (sand #1: KFU, N 25° 19’ 26.5404 E 49° 36’ 31.6692; and sand #4: Jleijlah, N 25° 28’ 52.9572 E 49° 35’ 23.1972), two nearby beaches on the sea (sand #2: Al-Oqair, N 25° 43’ 19.5636 E 50° 11’ 57.408), and a brackish water lake (sand #3: Al-Asfar lake, N 25° 31’ 7.3308 E 49° 47’ 51.486). The collected sand types were sterilized by heating at 280°C in a dry oven for at least 2 h. Fifty grams of one of the four sources of local sand were placed in incubation vials (200 mL, polypropylene). To test the effect of different directions of ventilation on larval growth, holes were made on the sides of the vials or on the lid, or the lids were replaced with gauze fixed with elastic bands. The collected larvae were individually placed in vials, with 10 replicates allocated for each incubation condition (four sand types and three ventilation methods: 12 different treatments, with 10 individually incubated larvae: 120 incubation vials). The type of bedding was represented using 30 larvae that were incubated in 10 vials (one, two, or three larvae in each vial) with no bedding. The 130 vials were incubated in an environmental chamber (HC30-2, Shell Lab, 28 Ft^3^, Cornelius, Oregon, USA) in complete darkness at a relative humidity (RH) of 60%–62% and temperatures ranging from 30°C to 36°C (Figure-1b). Another 25 vials containing sand type 3 (due to its abundance) were placed at room temperature (RT; 22°C–35°C) as negative controls (temperature ranged from 22°C to 35°C, RH ranged from 30% to 50%), 10 of the latter 25 vials contained a single L3, while the rest (15 vials) contained one to five larvae in each vial (totaled: 51 L3 in 15 vials).

### The critical weight needed for successful adult eclosion

L3 (n = 184) was collected from naturally infected camels. The weights of the larvae were obtained to the nearest 0.1 mg using a sensitive balance (KERN 870, Kern and Sohn, Albstadt, Germany). L3 was placed individually in vials containing sand types #1 or #3, with the lid replaced by a gauze covering (based on the results of the previous experiment of this study).

### Incubation conditions

**Figure-2:**
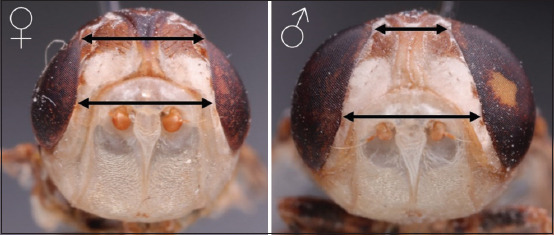
Heads of adult Cephalopina titillator flies. The female head (left) is characterized by eyes that are almost parallel to each other (indicated by the almost equal distances between the compound eyes, just above the antenna). The male head (right) has a broader space between the ventral sides of the compound eyes, as opposed to the dorsal side.

Each vial was inspected daily until the termination of the experiment at 30-day post-incubation (dpi). Pupariation was defined as the time elapsed from the onset of the transformation of the larvae into pupa, observed by the darkness of the larvae until the full darkening of the spines of the larvae (Figure-1c). The pupation period was defined as the time elapsed from the full darkness of the pupa until eclosion or the emergence of the imago or adult flies from the pupa (Figure-1d) [21]. The life span of flies was defined as the number of days adult flies were found viable, starting from the day of fly eclosion (emergence or hatching of puparium) until their death (lack of fly movement when stimulated by touching and carrying with a forceps). The following dimensions of the pupae were recorded: Length from anterior end to posterior end, width from one side to the other, and height from top to bottom (Figure-1c). Puparia that did not produce adult flies at the end of the experiment (30 dpi) were marked as non-viable and were cut open to examine their contents. The pupa that experienced eclosion was marked: viable. The RH and temperature (maximum and minimum) of the cabinet were monitored daily and recorded daily, starting with a 2-day pre-incubation period, and continued until the termination of the experiment. Sex determination of the emerged imagos was performed based on the shape and distance between the margins of the compound eyes (Figure-2) [13].

## Results

### Effect of the type of sand ground and ventilation direction

In this experiment, 17 adults emerged (five males and 12 females) out of 211 larvae were incubated in the environmental chamber or at RT, four of which were incubated at RT. Examination of the non-viable pupae at the end of the experiment showed either empty pupae (47.2%) or putrefied insect tissues (47.1%). Six of the 17 hatched adults were incubated in vials containing sand type 1 (KFU) and another six flies in sand type 3 (the beach of the nearby Al-Asfar Lake). The remaining flies (n = 3) emerged from vials incubated with sand #2 (the beach of a sea) and one incubated on sand type #4 (sand from a farm). No adult flies emerged from larvae incubated in vials without bedding. Eight of the 17 hatched flies (47%) were incubated in vials with gauze placed on top of the vial and fixed with elastic bands instead of the original plastic lid. Seven adults (41%) were incubated in vials with side holes, and the remaining two adults (12%) were incubated in vials with top holes.

### Weight of L3 for successful eclosion

The weights of 184 L3 larvae were obtained and ranged from 281 mg to 1.19 g. The minimum critical weight of L3 larvae required for successful eclosion was 754 mg (Table-1), and 89 larvae had at least the latter weight. Successful eclosion was recorded in 33 of the 89 larvae with the lowest critical weight (37.1%). No sex differences were observed in the minimum critical weight of mature L3 mice. The minimum critical weight of L3 for successful eclosion of male imagos was 947 mg (754 mg–1.19 g), whereas the minimum critical weight of L3 for successful eclosion of female imagos was 867 mg (762 mg–1.01 g). The male-to-female ratio of hatched flies was 19:14 (57.6% males, 42.4% females). At hatching, the weight of the flies ranged from 290 to 390 mg (averaged 352 mg) and decreased to 10–49 mg (averaged 19 mg) at the time adult flies died. On average, the emerged adults lost approximately 94.6% of their weight from the start of pupariation until death.

**Table-1:** Biological data of *in vitro* incubated third-stage larvae (L3) of *Cephalopina titillator* that produce adult flies.

Parameter	Data
Minimum critical weight of L3 for successful eclosion (all)	754 mg
Average and range of critical weight of L3 for successful eclosion (males)	947 mg (754 mg–1.19 g)
Average and range of critical weight of L3 for successful eclosion (females)	867 mg (762 mg–1.01 g)
Percentage of successful L3 eclosion with minimum critical weights	37.1% (33 of 89 L3)
Flies weight on the day of emergence	290–390 mg (averaged 352 mg)
Weight of flies at death	10–49 mg (averaged 19 mg)
Pupariation period (all)	1–9 days (averaged 3.6 days)
Pupariation period (males)	1–8 days (averaged 3.2 days)
Pupariation period (females)	1–9 days (averaged 4.4 days)
Pupation period (all)	5–13 days (averaged 10 days)
Pupation period (males)	6–12 days (averaged 10.1 days)
Pupation period (females)	5–13 days (averaged 9.4 days)
Life span of imagos (all)	3–18 days (averaged 10 days)
Life span of imagos (males)	3–16 days (averaged 8.1 days)
Life span of imagos (females)	6–18 days (averaged 12 days)

### Pupariation period and life span of adult flies

The pupariation of the emerged adults from both experiments (n = 45) took 1–9 days (averaged 3.6 days), and the pupation period lasted 5–13 days (averaged 10 days). There were no sex differences in pupariation and the pupation period. The pupation period of male flies averaged 3.2 days and ranged from 1 (n = 4) to 8 days (n = 3), whereas the pupation period in males averaged 10.1 days and ranged from 6 days (n = 3) to 12 days (n = 5). The pupation period of female flies averaged 4.4 days and ranged from 1 (n = 1) to 9 days (n = 1), whereas the pupation period in females averaged 9.4 days and ranged from 5 days (n = 1) to 13 days (n = 2).

The average life span of the emerged imagos at 30°C–36°C at 60%–62% RH was 10.1 days, ranging from 4 days (n = 1) to 18 days (n = 1). There was a tendency for female flies to survive longer or live longer than males. The average life span of males was 8.1 days, ranging from 3 days (n = 1) to 16 days (n = 2), whereas the average life span of females was 12 days, ranging from a minimum of 6 days (n = 1) and living up to 18 days (n = 1).

Several attempts were made to induce mating between emerged females and males by placing them together in the same vial in an environmental chamber or at RT. Mating events were observed and recorded, but egg/larva-laying events were not observed.

### Pupal dimensions

Regarding pupal dimensions, the viable pupae had an average length of 15.9 mm (ranging from 10 to 18 mm), an average width and height of 6.3 mm (ranging from 5 to 8 mm). The viable pupae of female flies were slightly longer but had lower height than their male counterparts. The viable pupae of female flies had an average length of 15.5 mm (ranging from 14 to 17 mm), average width of 5.8 mm (ranging from 5 to 7 mm), and average height of 5.3 mm (ranging from 5 to 7 mm). The viable pupae of male flies had an average length of 16.3 mm (ranging from 10 to 19 mm), an average width of 6.9 mm (ranging from 5 to 8 mm), and an average height of 7.2 mm (ranging from 6 to 8 mm). The dimensions of viable pupae overlapped in their ranges with those of non-viable pupae but, on average, had lesser dimensions. The non-viable pupae had an average length of 11 mm (ranging from 4 to 22 mm), an average width of 4.6 mm (ranging from 1 to 7 mm), and an average height of 2.9 mm (ranging from 1 to 7 mm). On examination of non-viable pupae, mummified flies were only found in three pupae, whereas the rest of the pupae had putrefied contents or were empty with no contents.

### RH and temperature

The study revealed that during the period of the experiment, the incubating chamber had an average low RH of 60.2% and high RH of 60.9% (ranging from 60 to 62%, SD: 0.45–0.64, respectively), and an average temperature of 33.4°C (ranging from 29.8°C to 36.2°C, SD 1.78).

## Discussion

To the best of the authors’ knowledge, this is the first study to report the minimum critical weight (754 mg) required for mature L3 of *C. titillator* to become an adult, with no sex differences in the L3 weight. In a similar study of another oestrid species, Cepeda-Palacios *et al*. [22] obtained a critical weight of 280 mg at which mature *Oestrus ovis* larvae become adult flies but reported sex differences in mature larval weight. The obvious difference in the critical weights of L3 between the two species and the presence of sex differences indicate the diversity of the biology among *Oestridae* flies.

A previous study by Khater [23] on *C. titillator* reported different eclosion success rates when incubating L3 under different conditions. An experimental study reported high eclosion success for *C. titillator* (86%–95%) when L3 was incubated at 26°C. Here, a reported eclosion rate of 37.1% was obtained when L3 stages were incubated at higher temperatures (30°C–36°C), which might indicate the adaptation of this species and its ability to survive hot conditions, but with a lower rate of fly emergence. The eclosion rates of the other oestrid flies; *Hypoderma lineatum* and *Hypoderma bovis*, were found most successful (86% and 72%, respectively) in alternating temperatures (15°C–25°C and 14°C–26°C and 16°C–24°C, respectively) than at constant temperatures (72% and 55%, respectively at 20°C) [24]. The selected temperatures in the latter study reflected the temperate habitats where larvae leave their hosts to pupate in relatively cold weather and when flies are most frequently found in nature [25, 26]. Epidemiological studies on the occurrence of *C. titillator* in Al-Ahsa, Saudi Arabia, revealed the presence of two peaks, one in February and another in September [5]. The temperature in the latter 2 months varied markedly, ranging from 8°C to 31°C in February and 20°C to 47°C in September [27]. Accordingly, the incubation temperatures employed in this study (30°C–36°C) highly resemble the ambient temperatures that the mature L3 of *C. titillator* may experience during the hot seasons. Nonetheless, testing the effect of incubating mature L3 of *C. titillator* at lower temperatures is encouraged to assess the success of eclosion during colder seasons of the year.

In the current study, most of the L3 incubated with *C. titillator* successfully formed puparium, but most of these pupae either did not complete pupal development or failed to emerge as adults. Mummified but fully grown adults were found inside three of the non-viable pupae (less than 2%) and the reason for their failed emergence was not known. Other studies on larvae of *O. ovis* have reported high mortality rates in wild populations, reaching up to 99% [21]. Mortality rates may be affected by abiotic and/or biotic factors. Further epidemiological studies are recommended to determine the mortality rates of immature stages of *C. titillator* and to determine their effects on population dynamics as well as the dispersion of infestations in camels.

Concerning the direction of ventilation of the vials, eclosion success was higher in vials with gauze replacing the lid and in vials with side holes. The authors noticed that most of the larvae submerged when they were placed in the vials and either pupated deep in the sand substrate or ascended and pupated on top of the sand. The results of this experiment suggest the marginal importance of the ventilation direction inside the vials, particularly if sufficient air exchange occurs between the interior environment of the incubating vials and the exterior. On the other hand, the presence of sand ground in the vials was essential for larvae to become adults, with a marked tendency toward higher eclosion success in vials with two specific types of sand: sand from inside the campus of KFU and from the beach of a nearby lake, in comparison to the other two types of sand from a farm and from the beach of the sea. In natural settings, sand is the type of ground in which the larvae of *C. titillator* are expected to pupate [20, 23]. The reported higher tendency of eclosion success in certain sand types might result from the physical and/or chemical characteristics of the sand itself [28].

In the pilot studies of this experiment, data were not shown; the authors experienced high mortality in larvae directly incubated without prior washing. Other similar studies on *C. titillator* excluded prior washing of the larvae but succeeded in obtaining viable imagos [20, 23]. The presence of blood stains on L3 might have interfered with the development of *C. titillator* in the current pilot studies by creating a favorable condition for bacterial growth, which may reflect the reduced survival of developing insects inside pupa [29].

The currently reported pupation period (5–13 days, averaged 9.8) at 30°C–36°C and RH of 60%–62% was found to be the shortest in relation to those previously reported from Saudi Arabia and other countries. According to a field and experimental study in Saudi Arabia [30], the researchers reported a pupation period of *C. titillator* of 15–20 days but did not state the employed temperature or RH. A report from Egypt [31] stated that at lower temperatures (20.8°C–25°C) but higher RH (75-85%) than those employed in the present study, the pupation period of *C. titillator* was variable and extended from 16 to 36 days. In Ethiopia [20], the recorded pupation period of *C. titillator* averaged 21 ± 1.4 days, also without stating the temperature and RH employed in their study. This variation in the pupation period might be explained by the currently employed high temperature (30°C–36°C), which resembles the environmental conditions in the hot seasons, whereas the previously reported longer pupation periods might resemble the conditions in the colder seasons.

The longevity of the flies in this study and that of the flies reported by Bekele [20] were comparable (12.4 ± 2.4 days), which might indicate the insignificance of the effect of the pupariation period on the life span of the emerged *C. titillator*. According to Rogers and Knapp [21], temperature, but not RH, highly regulates the eclosion rate of *O. ovis*. They also found that incubating L3 of *O. ovis* at constant temperatures did not result in adult emergence and that there was a threshold of fatal temperature (32°C) above which pupae died. Another study by Breev *et al*. [32] found that incubating L3 of *O. ovis* at low temperatures (20°C) reduced the mortality rate of pupae. These results support previous observations that suggested the adaptation of the life cycle of *C. titillator* to hot desert conditions, the abundance of larvae in its hosts, and higher infestation rates in colder seasons [5, 33].

## Conclusion

This study identified the minimum critical weight necessary for L3 to molt to the adult stage successfully. Depending on the source of the soil substrate, the success rate of adult eclosion from pupae was different, which might be an ecological factor contributing to the spread of *C. titillator* in nature. Manipulating the current *in vitro* conditions may help to model the emergence of *C. titillator* in different ecological settings worldwide.

## Authors’ Contributions

MNSA: Conceptualization, resources, methodology, formal analysis, and writing-original draft. HA, FA, and GA: Resources, methodology, investigation, and writing-review and editing. OA: Resources, writing, reviewing, and editing, and supervision. AM: Conceptualization, methodology, writing-review and editing, and supervision. All authors have read and approved the final manuscript.

## References

[1] Shamsi E, Radfar M.H, Nourollahifard S.R, Bamorovat M, Nasibi S, Fotoohi S, Hakimi Parizi M, Kheirandish R (2023). Nasopharyngeal myiasis due to *Cephalopina titillator* in Southeastern Iran:A prevalence, histopathological, and molecular assessment. J. Parasit. Dis.

[2] Safarov A, Kunisov B, Arepbaev I, Sazmand A (2024). First record of nasopharyngeal myiasis caused by *Cephalopina titillator* (Clark, 1816) in camel (*Camelus dromedarius* Linnaeus, 1758) in Uzbekistan. Vet. Parasitol. Reg. Stud. Reports.

[3] El-Hawagry M.S.A, Abdel-Dayem M.S, Dhafer H.M.A (2020). The family oestridae in Egypt and Saudi Arabia (Diptera, *Oestroidea*). Zookeys.

[4] Musa M.T, Harrison M, Ibrahim A.M, Taha T.O (1989). Observations on Sudanese camel nasal myiasis caused by the larvae of *Cephalopina titillator*. Rev. Elev. Med. Vet. Pays Trop.

[5] Fatani A, Hilali M (1994). Prevalence and monthly variations of the second and third instars of *Cephalopina titillator* (Diptera:*Oestridae*) infesting camels (*Camelus dromedarius*) in the Eastern Province of Saudi Arabia. Vet. Parasitol.

[6] Hussein M.F, Elamin F.M, El-Taib N.T, Basmaeil S.M (1982). The pathology of nasopharyngeal myiasis in Saudi Arabian camels (*Camelus dromedarius*). Vet. Parasitol.

[7] Yao H, Liu M, Ma W, Yue H, Su Z, Song R, Ma Q, Li L, Wu Z, Ma Y, Chen G, Chen B, Yang J (2022). Prevalence and pathology of *Cephalopina titillator*infestation in *Camelus bactrianus* from Xinjiang, China. BMC Vet. Res.

[8] Khater H.F, Ramadan M.Y, Mageid A.D (2013). *In vitro* control of the camel nasal botfly, *Cephalopina titillator*, with doramectin, lavender, camphor, and onion oils. Parasitol. Res.

[9] Attia M.M, Farag H.S, Abdel-Saeed H, Ismael E (2020). Advanced immunological studies on *Cephalopina titillator*with special references to the epidemiological uses of Dot-ELISA in camel sera. J. Parasit. Dis.

[10] Hassan N.M.F, Sedky D, Ezz N, Shanawany E.E.E (2022). Seroprevalence of nasal myiasis in camels determined by indirect enzyme-linked immunosorbent assay utilizing the most diagnostic *Cephalopina titillator* larval antigens. Vet. World.

[11] Aboelsoued D, Toaleb N.I, Mohamed A.M, Abdel Megeed K.N, Hekal S.H.A (2024). Serodiagnosis of nasal myasis in camels (*Camelus dromedaries*) in Egypt using third larval instar affinity-purified glycoprotein. Vet. Res. Commun.

[12] Higgins A.J (1985). Common ectoparasites of the camel and their control. Br. Vet. J.

[13] Zumpt F (1965). Myiasis in Man and Animals in the Old World.

[14] Nogueira S.N.L, Da Silva M.F, Furtado R.A, Paulino Júnior D, Soares M.C, Andrade M.M.A, Nascimento E.G, Ferreira L.L, Soares V.E, Lopes W.D.Z, Paranhos de Mendonça R (2020). *In vitro* test for the evaluation of the efficacy of topical products for the control of *Cochliomyia hominivorax* larvae. Parasitology.

[15] Medeiros M.T, Campos D.R, Soares E, Assis J.D, De Oliveira G.F, Santos L.O, Silva T.M.E, Da Silva M.P.D, Cid Y.P, Scott F.B, Comendouros K (2023). Larvicidal activity *in vitro* of essential oils against *Cochliomyia hominivorax*. Vet. Parasitol.

[16] Kotze A.C, Bagnall N.H, Ruffell A.P, George S.D, Rolls N.M (2022). Resistance to dicyclanil and imidacloprid in the sheep blowfly, *Lucilia cuprina*, in Australia. Pest Manag. Sci.

[17] Jia Z, Hasi S, Zhan D, Vogl C, Burger P.A (2024). Transcriptomic profiling of different developmental stages reveals parasitic strategies of *Wohlfahrtia magnifica*, a myiasis-causing flesh fly. BMC Genomics.

[18] Hussein M.F, Hassan H.A, Bilal H.K, Basmae'il S.M, Younis T.M, Al-Motlaq A.A, Al-Sheikh M.A (1983). *Cephalopina titillator* (Clark 1797) infection in Saudi Arabian camels. Zentralbl. Veterinarmed. B.

[19] Zayed A.A (1998). Localization and migration route of *Cephalopina titillator* (Diptera:*Oestridae*) larvae in the head of infested camels (*Camelus dromedarius*). Vet. Parasitol.

[20] Bekele T (2001). Studies on *Cephalopina titillator*, the cause of 'Sengale'in camels (*Camelus dromedarius*) in semi-arid areas of Somali State, Ethiopia. Trop. Anim. Health Prod.

[21] Rogers C, Knapp F (1973). Bionomics of the sheep bot fly, *Oestrus Ovis*. Environ. Entomol.

[22] Cepeda-Palacios R, Frugère S, Dorchies P (2000). Expected effects of reducing *Oestrus ovis* L. mature larval weight on adult populations. Vet. Parasitol.

[23] Khater H.F (2014). Bioactivities of some essential oils against the camel nasal botfly, *Cephalopina titillator*. Parasitol. Res.

[24] Pfadt R.E, Lloyd J.E, Sharafi G (1975). Pupal development of cattle grubs at constant and alternating temperatures. J. Econ. Entomol.

[25] Pfadt R.E (1947). Effects of temperature and humidity on larval and pupal stages of the common cattle grub. J. Econ. Entomol.

[26] Salt R.W (1944). The effect of subzero temperatures on *Hypoderma lineatum*. Devill. Sci. Agric.

[27] Ministry of Defence and Aviation K.O.S.A (2022). National Meteorology and Environment Centre:Surface Annual Climatological Report.

[28] Simco J.S, Lancaster L.J (1964). Effects of soil type, moisture level, and temperature on larval and pupal stages of the common cattle grub, *Hypoderma lineatum*. J. Kans. Entomol. Soc.

[29] Duranton C, Dorchies P (1997). *In vitro* culture of *Oestrus ovis* (Linné1761) first instar larvae:Its application to antiparasitic drug screening. Int. J. Parasitol.

[30] Banaja A.A, Madbouly M.M (1981). Field and laboratory observations on three dipterous larvae causing myiasis in livestock in the Western Region of Saudi Arabia. Bull. Fac. Sci.

[31] Fahmy M, Ashmawy K, Omar H.M (1985). Studies on the pupation period of the masal bot fly of camel (*Cephalopina titillator* Clarck, 1797) in Egypt. J. Egypt. Vet. Med. Assoc.

[32] Breev K.A, Zagretdinov R.G, Minár J (1980). Influence of constant and variable temperatures on pupal development of the sheep bot fly (*Oestrus ovis* L.). Folia Parasitol. (*Praha*.

[33] Al-jindeel T, Jasem H, Alsalih N, Al-Yasari A (2018). Clinical, immunological and epidemiological studies of nasopharyngeal myiasis in camels slaughtered in Al-Muthanna province. Adv. Anim. Vet. Sci.

